# Assessment of valgus laxity after release of the medial structure in medial open-wedge high tibial osteotomy: an in vivo biomechanical study using quantitative valgus stress radiography

**DOI:** 10.1186/s12891-019-2859-7

**Published:** 2019-10-27

**Authors:** Dai Sato, Eiji Kondo, Koji Yabuuchi, Jun Onodera, Tomohiro Onodera, Tomonori Yagi, Keita Sakamoto, Akira Takasawa, Norimasa Iwasaki, Kazunori Yasuda

**Affiliations:** 1 Department of Orthopedic Surgery, Faculty of Medicine and Graduate School of Medicine, Hokkaido University, Kita-15 Nishi-7, Kita-ku, Sapporo, 060-8638 Japan; 2 Centre for Sports Medicine, Hokkaido University Hospital, Kita-14 Nishi-5, Kita-ku, Sapporo, 060-8648 Japan; 3 Department Orthopedic Surgery, Yagi Orthopedic Hospital, 1-35, Nishino-3-5, Nishi-ku, Sapporo, 063-0033 Japan; 4 Department of Diagnostic and Interventional Radiology, Hokkaido University Hospital, Kita-14 Nishi-5, Kita-ku, Sapporo, 060-8648 Japan; 5 Department of Pathology, Sapporo Medical University School of Medicine, S-1 W-17, Chuo-ku, Sapporo, 060-8556 Japan

**Keywords:** Medial open-wedge high tibial osteotomy, Superficial layer of the medial collateral ligament, Valgus instability, Valgus stress test

## Abstract

**Background:**

To perform medial open-wedge high tibial osteotomy (OWHTO), surgeons expose the medial-proximal tibia by releasing or cutting the superficial layer of the medial collateral ligament (sMCL). Biomechanically, the sMCL provides primary restraint against valgus forces. Therefore, any release of the sMCL can cause valgus instability of the knee joint. The purpose of this study was to assess valgus laxity after release of the medial structure of the knee during OWHTO.

**Methods:**

Between 2009 and 2015, 84 consecutive patients (93 knees) who underwent OWHTO using a locking plate were enrolled in this study. All patients underwent radiological examinations before surgery, during surgery, 1 year after surgery, and after plate removal to objectively assess valgus laxity. The medial joint space (MJS) and the joint line convergence angle (JLCA) of the knee were evaluated using quantitative valgus stress radiography. Clinical evaluation was performed 2 years after surgery.

**Results:**

The mean functional knee score improved significantly, from 65.5 to 91.1 points (*p* < 0.0001). The mechanical axis percentage shifted to pass through a point 69.7% lateral from the medial edge of the tibial plateau. The MJS and JLCA increased significantly during OWHTO surgery (11.0 mm, 7.4 °, *p* < 0.0001). However, no significant differences were noted in the MJS and JLCA among preoperative, 1-year postoperative periods and after plate removal.

**Conclusion:**

Valgus laxity was significantly greater after release of the sMCL. However, no significant differences were noted in valgus laxity in preoperative, 1-year postoperative periods and after plate removal. Complete release of the sMCL did not cause postoperative valgus laxity after OWHTO surgery.

**Trial registration:**

Trial registration number: No.012–0360.

## Introduction

High tibial osteotomy (HTO) has been a useful surgical option for medial osteoarthritis (OA) and spontaneous osteonecrosis of the knee (SONK) [1, 2]. Recently, medial open-wedge (OW) HTO with a locking plate has attracted a great deal of attention [1–9]. To obtain sufficient opening of the medial side of the proximal tibia in OWHTO, surgeons should detach the distal attachment of the superficial layer of the medial collateral ligament (sMCL) or completely cut the sMCL at the osteotomy level [10]. The human MCL consists of three units: the superficial parallel-oriented fibers (sMCL), the deep medial capsular ligament, and the posterior oblique ligament [11–13]. Biomechanically, the sMCL is the primary restraint to valgus forces and plays a significant role in restraining external rotation [14, 15]. Therefore, detachment or cut of the sMCL induces valgus instability of the knee joint [11, 16–19]. Clinically, it is known that valgus instability of the knee results in a serious disturbance in walking [20]. Even for aged patients, Dragosloveanu et al. [21] reported that repair of collateral ligament injuries must be performed during surgery, especially complete ruptures of the MCL. Therefore, valgus instability of the knee due to detachment or cut of the sMCL is a potential problem in OWHTO, although such valgus instability has not been included in a list of common complications following OWHTO. Pape et al. [19] suggested that the release of the sMCL should be kept to a minimum to decrease the potential for valgus instability following OWHTO, based on their basic biomechanical study.

Most recently, Seo et al. [22] reported the first clinical study to evaluate the changes in valgus laxity of the knee joint after OWHTO with a locking plate using valgus stress radiographs. They reported that complete release of the sMCL increased valgus laxity during surgery, but the laxity decreased to the level before sMCL release at 3 and 12 months. However, they did not measure valgus laxity after removal of the locking plate, which might affect the measured valgus laxity as late instability. In addition, their clinical outcomes were still lacking. Thus, the database on valgus laxity of the knee after OWHTO is still insufficient. Specifically, it remains unclear how much valgus laxity remains after removal of the medial locking plate.

The purpose of present study was to investigate the changes in the valgus laxity of the knee joint after removal of the medially placed locking plate in patients who underwent OWHTO with complete release of the sMCL using quantitative valgus stress radiography. The hypotheses were that (1) release of the distal attachment of the sMCL may significantly increase valgus laxity immediately during surgery; (2) there may be no significant difference in valgus laxity between pre-operative and at 1 year periods after OWHTO with medial locking plate fixation; and (3) removal of the locking late after OWHTO may not significantly increase valgus laxity.

## Materials and methods

### Study design

Between January 2009 and July 2015, a prospective cohort study was performed with 84 consecutive patients (93 knees) who underwent OWHTO with a locking plate (TomoFix® Medial High Tibial Plate, DepuySynthes, West Chester, PA, USA, or TriS Medial HTO Plate System, Olympus Terumo Biomaterials, Tokyo, Japan). Inclusion criteria involved persistent pain due to medial compartment OA or the SONK of medial femoral condyle. Each patient received conservative treatment for at least 3 months. Exclusion criteria included: (1) lateral femorotibial angle (FTA) greater than 185°; (2) loss of knee extension more than 15°; (3) range of knee motion less than 130°; (4) history of infection in the knee; (5) severe OA in the patellofemoral or/and the lateral femorotibial joints; and (6) cruciate ligament insufficiency or varus/valgus instability of greater than 10°. All patients underwent clinical and radiological examinations before surgery and 2 years after surgery. In addition, 10 patients underwent spot MRI evaluations before surgery and after plate removal. In three patients, a needle biopsy was taken from the distal attachment of the sMCL before surgery and after plate removal.

### Patient demographics

The overall demographic data are summarized in Table 1. There were 20 men and 64 women with a mean age of 60.4 (range 41–72) years at the time of surgery. Overall, 84 knees were diagnosed as medial compartment OA, and 9 knees were diagnosed as SONK of the medial femoral condyle.

**Table 1:** Patients’ background characteristics

Variable	Frequency
Age (y)	60.4 (8.7)
Male/female (patients)	20/64
Right/left side (knees)	51/42
Unilateral/bilateral (knees)	84/9
Body weight (kg)	65.7 (12.1)
BMI (kg/m^2^)	26.6 (4.0)
Young adult mean (%) of the bone mineral density	90.8 (15.8)
OA grade^a^ (patients)	
Grade 0	0 knees
Grade 1	0 knees
Grade 2	17 knees
Grade 3	61 knees
Grade 4	6 knees
SONK^b^ (patients)	
Stage 1	0 knees
Stage 2	4 knees
Stage 3	4 knees
Stage 4	1 knee

Mean (standard deviation).

*OA* osteoarthritis, *SONK* spontaneous osteonecrosis of the knee.

^a^Kellgren-Lawrence grading system

^b^Koshino’s stage [23]

### Preoperative planning

Preoperative planning with an appropriate correction angle of the tibia is made using a standing full-length lower limb anteroposterior radiograph [24]. OW osteotomy lines are drawn on the full-length lower limb radiograph so that the hinge point, P, is located at approximately 5 mm medial from the tibio-fibular joint on the lateral tibial condyle. To calculate an appropriate angle of the medial opening wedge, a long line, [A], is drawn from the center of the femoral head through the 65% lateral from the medial edge of the tibial plateau on the lateral tibial plateau. Then, another line, [B], is drawn from the hinge point [P] to the center of the talar dome, and the length of line B is measured. Then, an arc, [C], the center and the radius of which are the hinge point P and line B, respectively, is drawn so that the arc is across line A. A line, [D], is drawn from the hinge point P to the crossing point between line A and the arc C. Then, the angle formed between line B and line D provides the medial opening angle, which is identical to the correction angle of the lower limb alignment. A medial opening line from the hinge point [P] is drawn using this angle. Finally, the medial opening distance is also measured on the medial side of the proximal tibia.

### Surgical procedure of OWHTO

**Fig. 1:**
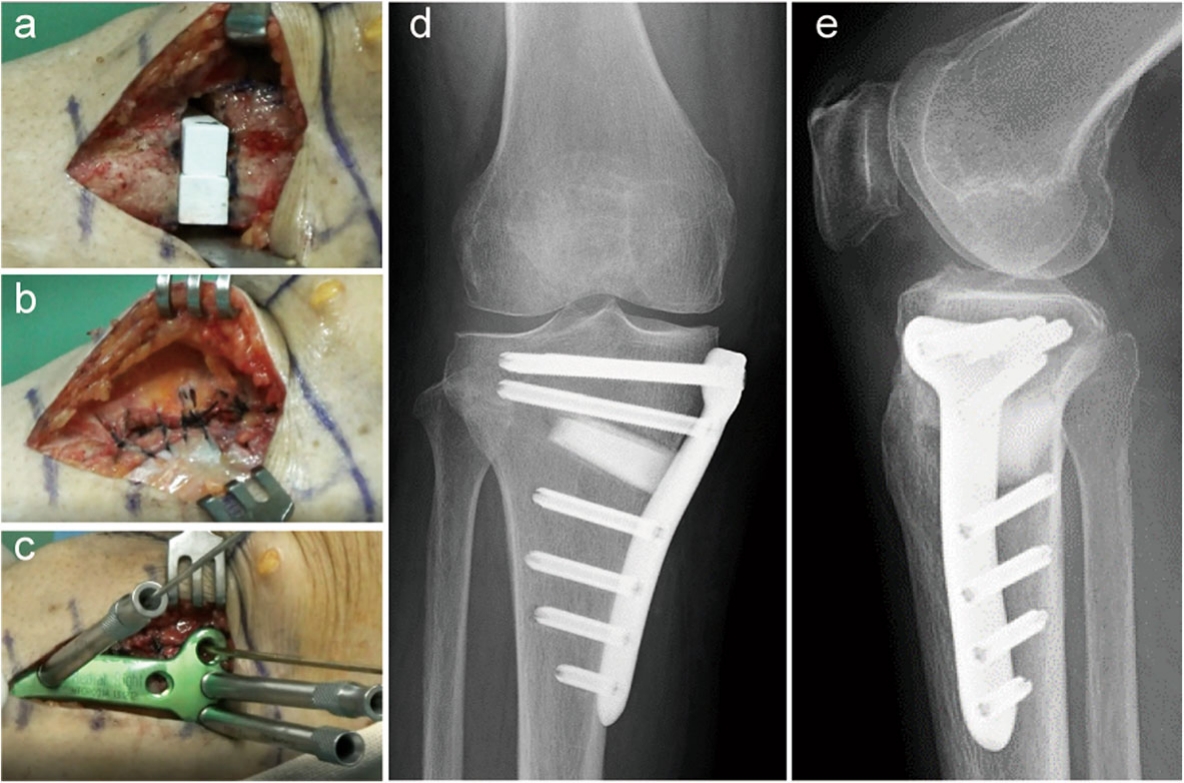
**a** Equivalent to the size of the opening, the wedged β-tricalcium phosphate spacers (Osferion60, Olympus Terumo Biomaterials) are inserted into the osteotomy site to fit into the medial cortical bone edge. **b** sMCL, pes anserinus, and periosteum are repaired. **c** The locking plate (TriS Medial HTO Plate System, Olympus Terumo Biomaterials) is fixed on the medial aspect of the proximal tibia. Postoperative anteroposterior (**d)** and lateral (**e)** radiographs of the right knee show a low-profile locking plate (TriS Medial HTO Plate System, Olympus Terumo Biomaterials) fixed in place with eight locking screws

Before OWHTO, arthroscopy was routinely performed to evaluate the cartilages, menisci, and cruciate ligaments. Additional treatments of the intra-articular lesions are shown in Table 2. The OWHTO procedure was recently reported in detail [25]. A 7-cm medial longitudinal incision was made in the proximal tibia. Complete release of the medial structure of the knee was performed, which consisted of the distal attachment of the sMCL, the pes anserinus, and periosteum. Then, an ascending biplanar osteotomy of the tibial tuberosity was performed. The oblique osteotomy site was gradually opened for the correction angle and target width using a protractor-installed specially designed spreader (Olympus Terumo Biomaterials) based on preoperative planning. Under fluoroscopic control, the surgeon confirmed that the mechanical axis of the correct lower limb using a long straight metal rod. When the mechanical axis (MA) passed through a point 65% lateral from the medial edge of the tibial plateau, the wedged β-tricalcium phosphate spacers (Osferion60, Olympus Terumo Biomaterials) were implanted parallel into the posterior opening space (Fig.1a). Before implantation of the plate, the sMCL, pes anserinus, and periosteum were sutured using absorbable sutures (Fig. 1b). Finally, the tibia was fixed with a locking plate (Fig. 1c) by inserting 8 locking screws (Fig. 1d, e).

**Table 2:** Arthroscopic intra-articular treatment

	Number of knees
Partial meniscectomy	
Medial meniscus	69 knees
Lateral meniscus	13 knees
Microfracture	41 knees
Osteochondral autograft transfer	9 knees

### Clinical and radiological evaluations

The patients were evaluated using the functional knee scoring scale (Japanese Orthopaedic Association (JOA) score for osteoarthritic knees) [26, 27]. The clinical evaluations were carried out twice, before surgery and 2 years after OWHTO. Radiographs were obtained both pre-operatively and postoperatively (one and 2 years after OWHTO). The FTA was measured on an anteroposterior weight-bearing radiograph of a single leg with the knee joint in extension. The MA percentages, the hip knee angle (HKA), and the medial proximal tibial angle (MPTA) were measured on an anteroposterior radiograph of the whole lower limb taken with a long cassette in the one-leg standing position. The Insall–Salvati index [28] and posterior tibial slope (PTS) [29] were estimated on lateral radiographs.

### Valgus laxity evaluation

**Fig. 2:**
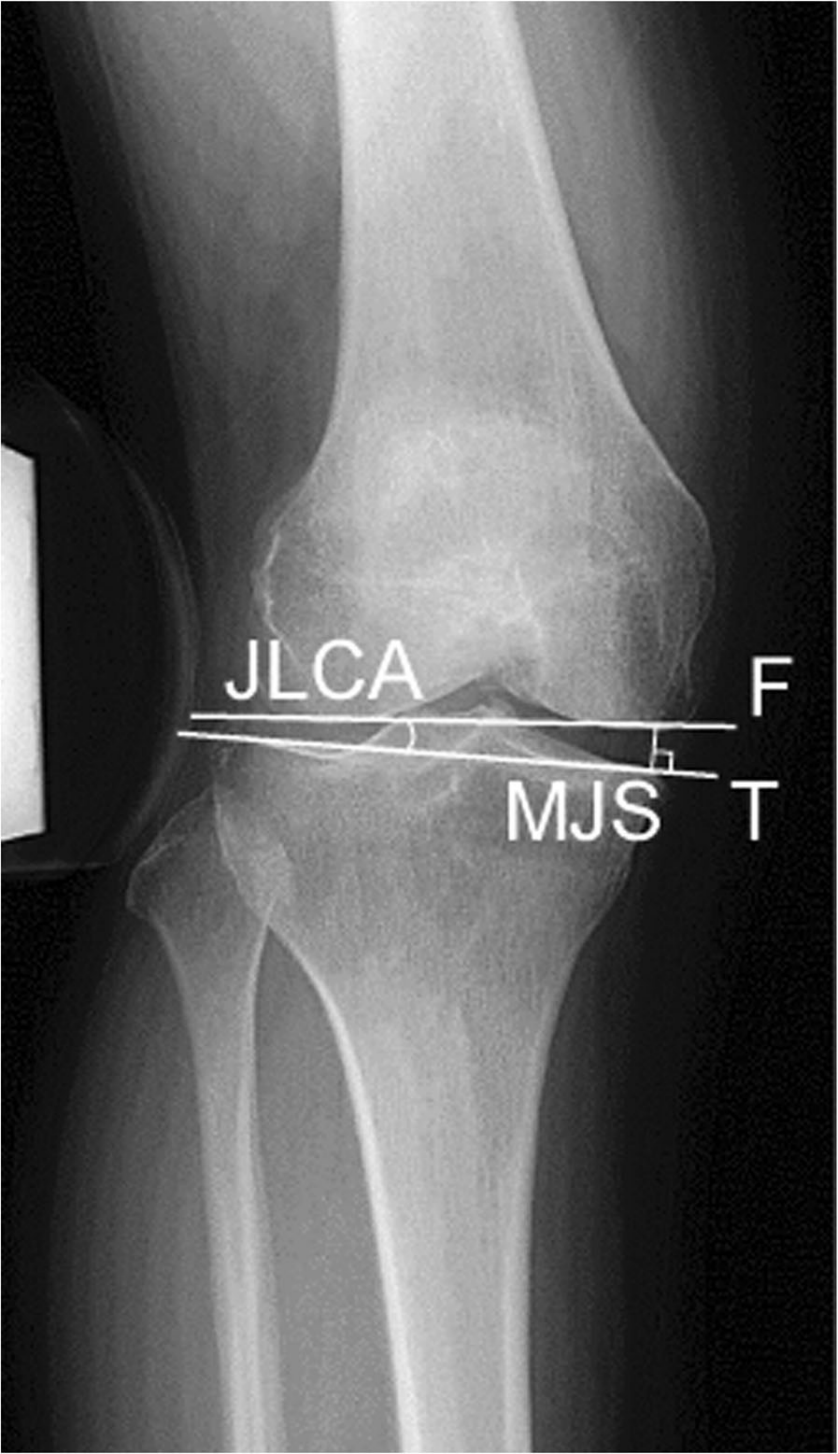
Measurement of the medial joint space (MJS) and joint line convergence angle (JLCA)

To objectively assess valgus laxity, the joint line convergence angle (JLCA) and the distance of the medial joint space (MJS) were evaluated with a 150-N valgus force at 20° of knee flexion using telos device (Metax, Hungen-Obbornhofen, Germany). Anteroposterior stress radiographs were obtained five times: 1) before surgery; 2) before releasing the distal attachment of the sMCL under general anesthesia; 3) after releasing the sMCL; 4) 1 year after OWHTO surgery; and 5) 2 years after OWHTO surgery (after plate removal). Then, intraoperative valgus stress radiographs were performed using a knee laxity tester (Kalamazoo, MI, USA). The distance of the MJS [30] was calculated according to the method reported by Sawant et al. (Fig. 2). The JLCA [31] was calculated according to the method reported by Lee et al. (Fig. 2).

### MRI evaluation

Ten patients underwent spot MRI evaluations before surgery and after plate removal. MRI was performed using a 1.5-Tesla whole-body clinical scanner (Achieva TX-series; Philips Healthcare, Best, The Netherlands). The distal attachment of the sMCL of each subject was centered in a microscopy coil in the supine position with the knee slightly flexed. Two-plane (coronal and sagittal) localizer imaging was performed for the distal attachment of the sMCL. The parameters for this imaging included: repetition time (TR), 4000–8000 ms; echo time (TE), 70 ms; matrix size, 256 × 256; slice thickness, 2 mm; and number of 16 slices.

### Histological evaluation

Samples of the distal attachment of the sMCL were taken to use a biopsy needle. The samples were fixed with 10% formalin, dehydrated, embedded in paraffin, and sliced into 4-μm-thick sections stained with hematoxylin and eosin (H-E), Masson trichrome, and Elastica van Gieson (EVG).

### Statistical analysis

All data are shown as means with standard deviations. A commercially available software program (GraphPad Software, Inc., San Diego, CA, USA) was used for the statistical calculations. The paired *t-*test was used to assess the clinical and radiological differences between before and after surgery. Repeated-measures analysis of variance (ANOVA) was used to compare valgus instability among the five groups: 1) before surgery; 2) before releasing the proximal tibial attachment the sMCL under general anesthesia; 3) after releasing the proximal tibial attachment of the sMCL; 4) 1 year after surgery; and 5) 2 years after surgery (after plate removal). To identify which preoperative, intraoperative, and postoperative factors affect knee laxity in the valgus stress test and other related outcomes, Pearson correlation analysis was used. The significance level was set at *p* = 0.05.

## Results

### Overall clinical and radiological outcomes

The mean opening angle and distance were 10.1 ± 3.5° and 11.9 ± 2.6 mm. The mean JOA score improved significantly from 65.5 points to 91.1 points (Table 3). The mean FTA and HKA changed significantly from 179.6° to 169.6° and from − 4.5° to 3.7°. The mean MA shifted to a point 69.7% lateral from the medial edge of the tibial plateau. The mean MPTA and tibial slope angle were significantly increased from 85.5° to 93.3° and from 8.5° to 11.0°.

**Table 3:** Comparisons of clinical and radiological outcomes between pre-operative and 2 years postoperative

	Pre-operative	2 years postoperative	*p* value^a^
JOA score (points)	65.5 (10.1)	91.1 (8.3)	< 0.0001
FTA (°)	179.6 (3.3)	169.6 (2.3)	< 0.0001
HKA (°)	−4.5 (2.9)	3.7 (1.9)	< 0.0001
MA (%)	27.4 (12.6)	69.7 (13.0)	< 0.0001
MPTA (°)	85.5 (2.8)	93.3 (2.5)	< 0.0001
Insall-Salvati ratio (%)	0.9 (0.1)	0.9 (0.1)	N.S.
PTS (°)	8.5 (3.2)	11.0 (3.4)	< 0.0001

*JOA score* Japanese Orthopaedic Association score for osteoarthritic knee, *FTA* lateral femorotibial angle, *HKA* hip knee angle, *MA* mechanical axis, *MPTA* medial proximal tibial angle, *PTS* posterior tibial slope.

^a^Significance levels with Student’s *t*-test

### Analysis of valgus laxity

**Fig. 3:**
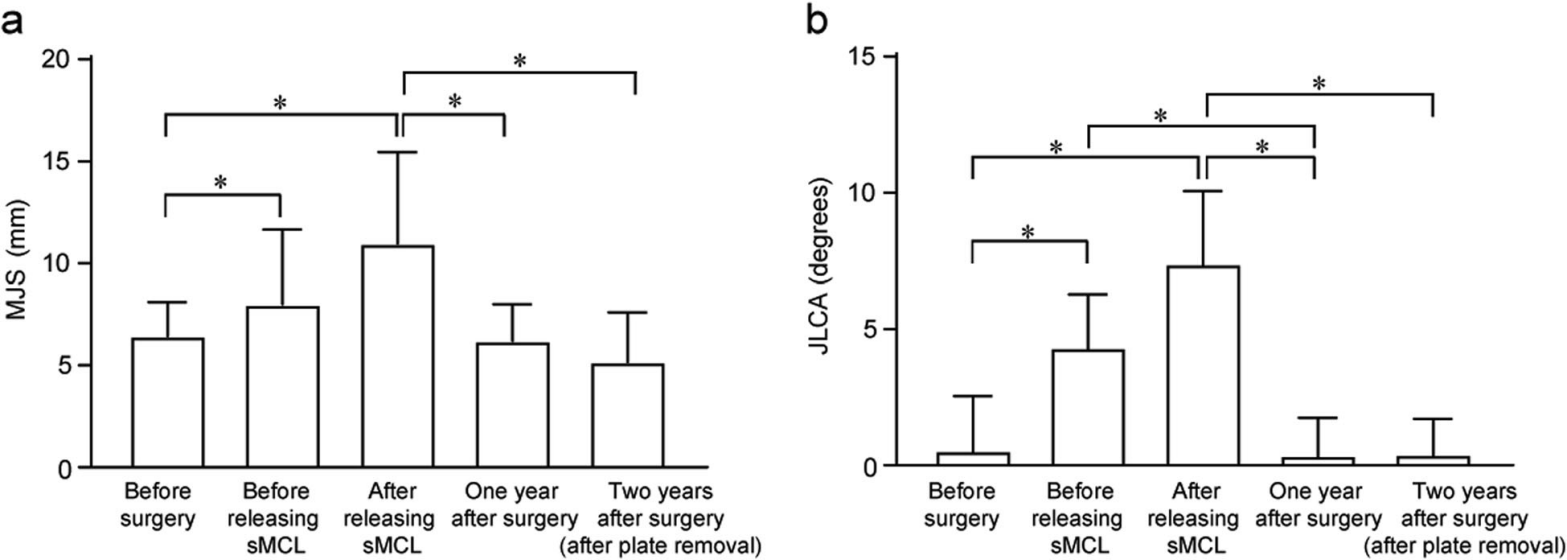
Changes in the medial joint space (MJS) (**a**) and in the joint line convergence angle (JLCA) (**b**) in all patients. Error bars represent the standard deviation. *Statistically significant difference (*p* < 0.05)

**Fig. 4:**
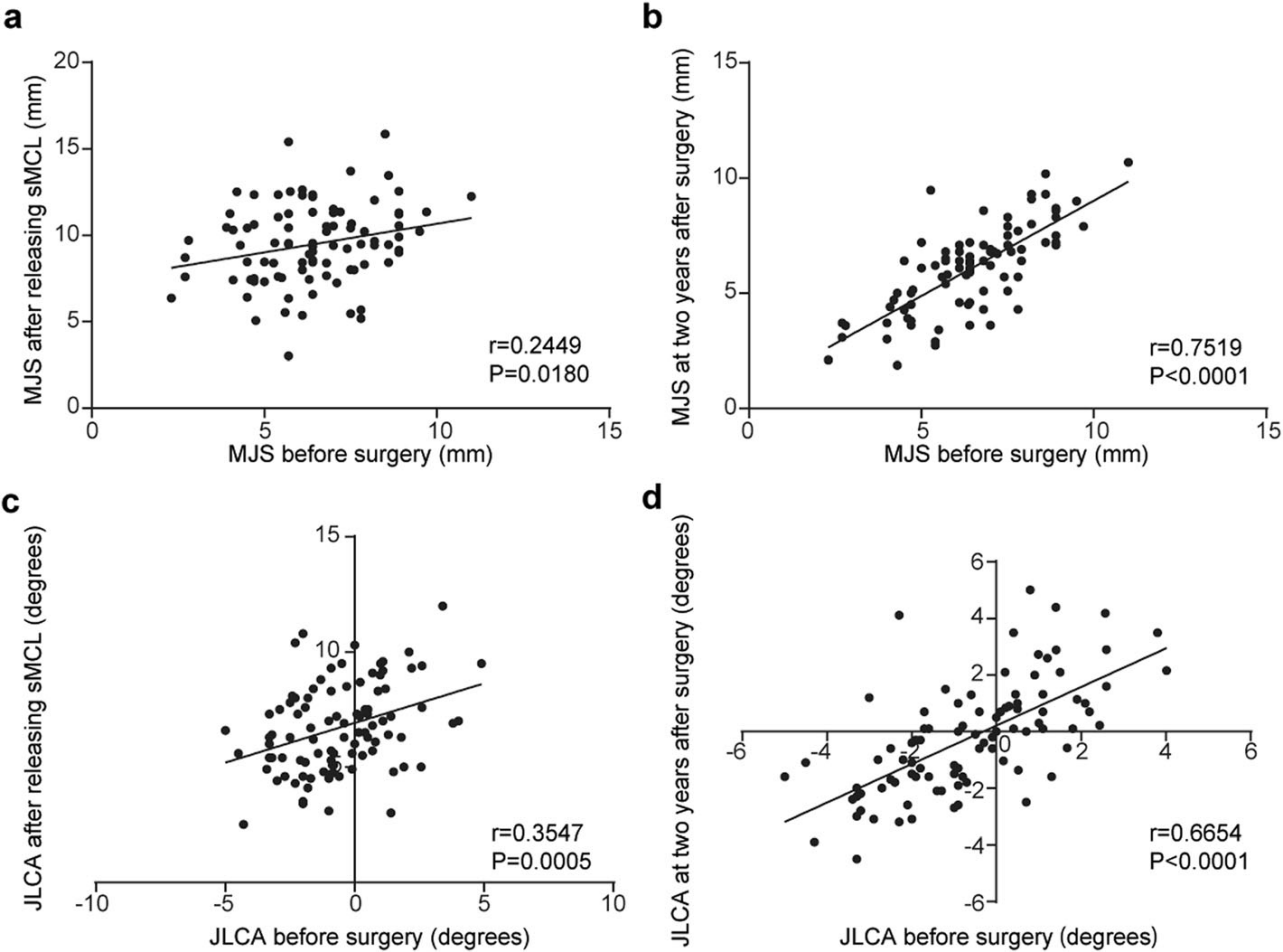
Pearson correlation analysis shows that the MJS and JLCA before surgery are significantly correlated with the MJS (*r* = − 0.2449, *p* = 0.0180) and JLCA (*r* = − 0.3547, *p* = 0.0005) after releasing the sMCL (**a**, **c**) and the MJS (*r* = − 0.7519, *p* < 0.0001) and JLCA (*r* = 0.6654, *p* < 0.0001) after plate removal (2 years after OWHTO) (**b**, **d**)

**Fig. 5:**
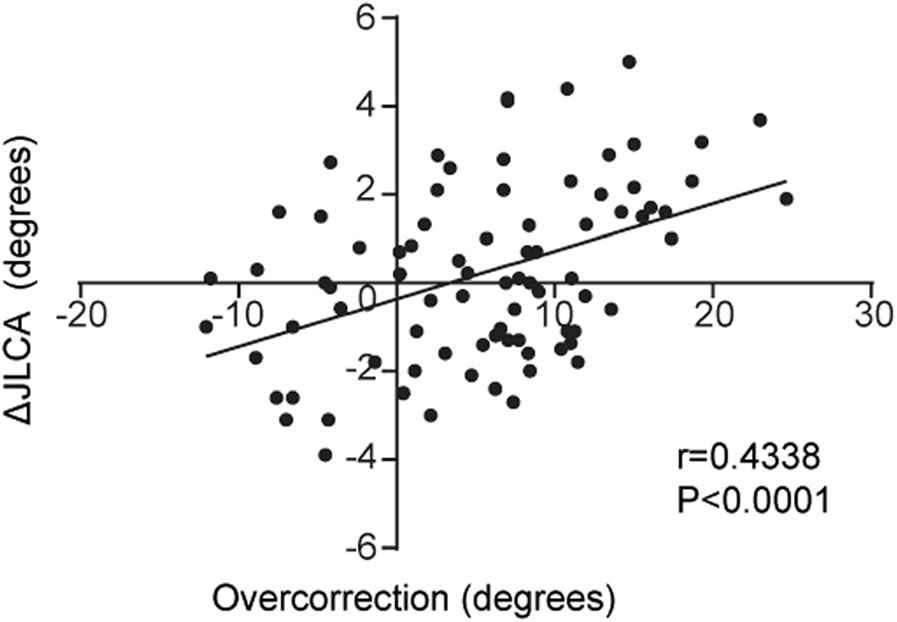
The relationship between ΔJLCA and overcorrection. The scatter plot shows a moderate correlation between ΔJLCA and overcorrection (*R* = 0.4338)

Comparisons of MJS and JLCA among 5 periods are presented in Fig. 3a, b. The MJS and JLCA before releasing the medial structure under general anesthesia was significantly increased compared with the MJS and JLCA before surgery (6.4 ± 1.7 mm to 8.0 ± 3.7 mm and − 0.5 ± 2.0° to 4.3 ± 1.9°). In addition, the MJS and JLCA after release of the medial structure during surgery was significantly increased (6.4 ± 1.7 mm to 11.0 ± 4.5 mm and − 0.5 ± 2.0 ° to 7.4 ± 2.7°). However, there were no significant differences in the MJS and JLCA among the three periods before surgery, 1 year after OWHTO surgery, and after plate removal (Fig. 3). The correlation analysis demonstrated that the MJS and JLCA before surgery were moderately correlated and significantly more than those after sMCL release (*p* < 0.0180) and after plate removal (*p* < 0.0001) (Fig. 4). The regression analysis showed moderate correlation between ΔJLCA (preoperative JLCA - postoperative JLCA) and overcorrection (postoperative MA - 65%) [correlation coefficient (R), 0.4338] (Fig. 5).

### MRI evaluation

**Fig. 6:**
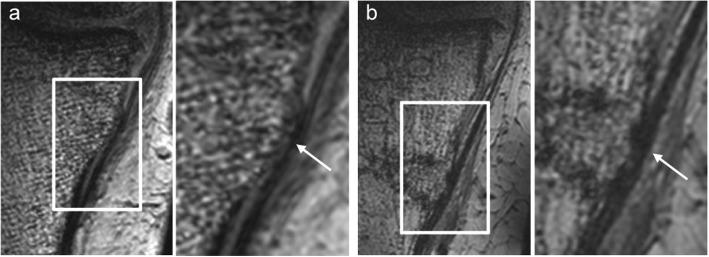
**a** Preoperative T2-weighted coronal plane MRI showing the sMCL. **b** After removal of a locking plate, the sMCL (indicated by white arrows) shows iso-intensity and is thicker than on the preoperative image

Spot MRI showed that the sMCL was clearly seen as regular in shape before OWHTO surgery. On the other hand, after OWHTO, the MRI showed that the sMCL was meandering and seen to be thicker with iso-intensity compared to before surgery (Fig. 6).

### Histological evaluation

**Fig. 7:**
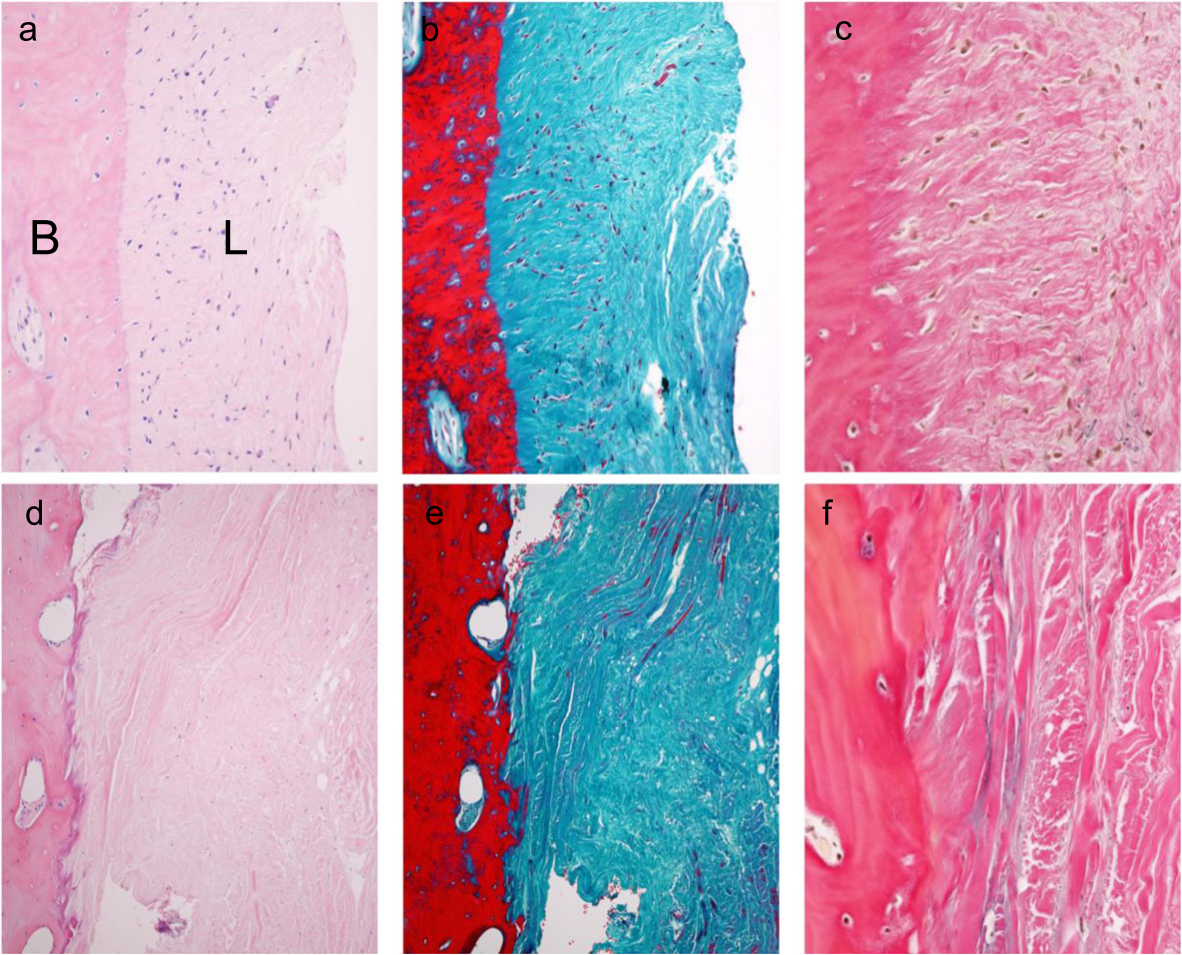
Images showing the histomorphometry using hematoxylin-eosin (left), Masson trichrome (middle) and Elastica van Gieson (right) staining of the distal attachment of the sMCL in the normal control **a**, **b**, 100x magnified, **c** 400x magnified. After removal of a locking plate **d**, **e**, 100x magnified, **e** 400x magnified. L: ligament B: bone

In the normal control, H-E staining showed many cell nuclei in the distal attachment of the sMCL (Fig. 7a). Masson trichrome and EVG staining showed intact elastic fibers in the bone in the sMCL (Fig. 7b, c). On the other hand, after HTO surgery, H-E staining showed signs of necrotic cell nuclei in the sMCL and bone (Fig. 7d). The bone-ligament interface was filled with unstructured fibrous connective tissue. On Masson trichrome and EVG staining, collagen fiber bundles entering into the bone substrate were irregularly oriented (Fig. 7e, f).

## Discussion

The most important findings of the present study were that: (1) the release of the distal attachment of the sMCL significantly increased valgus laxity immediately during OWHTO surgery; (2) there was no significant difference in the valgus laxity between the pre-operative and at 1 year periods after OWHTO with medial locking plate fixation; and (3) removal of the locking plate after OWHTO did not significantly increase valgus laxity. These results suggest that release of the distal attachment of the sMCL may not cause valgus laxity after OWHTO surgery.

In several biomechanical studies, release of the sMCL is essential for osteotomy procedure when performing OWHTO [2, 10, 19, 32]. There studies also reported that this procedure is necessary to avoid not only an increase in medial joint pressure and posterior slope, but also neurovascular injury by inserting a protector to the posterior tibia during surgery. First, decompression of the medial compartment, as the main goal of OWHTO surgery, cannot be achieved if the sMCL remains intact, because the medial opening space is created between the proximal and the distal attachment of the sMCL. Agneskirchner et al. [10] tested human knee specimens with a load of 1000 N with different loading alignments in extension using a material testing machine. They concluded that complete release of the distal fibers of the sMCL is necessary to decompress medial joint pressure after OWHTO. Second, the tibial slope increase in OWHTO was caused by asymmetric opening of the oblique osteotomy site [2]. If the osteotomy is started in the medial tibial head (anterior to the MCL), the osteotomy is too short in the posterior part of the tibia. Then, the posterior part of the tibial plateau is unavoidably lifted less than the anterior part, and the slope is thus increased. Therefore, the sMCL should be released to avoid increasing the tibial slope in OWHTO, as reported by Lobenhoffer et al. [2]. Third, protection for the posterior neurovascular structure is necessary during wedge osteotomy from the medial cortex of the tibia to the proximal tibiofibular joint in OWHTO. Previous studies reported that the frequency of popliteal artery injury was 0.4–1.7% during OWHTO [33–35]. Attinger et al. [32] reported that sufficient medial exposure for orientation and protection by releasing the sMCL during OWHTO surgery is mandatory for a safe osteotomy. In their report, it was easy to insert a blunt retractor to protect the popliteal artery when releasing the sMCL.

Previous basic studies reported that the sMCL is the ‘primary’ restraint to valgus forces [11, 13, 15, 18, 19, 36]. Seo et al. [22] reported that a statistically significant difference was observed between the MJS before (mean; 9.0 mm) and after release of the sMCL (12.2 mm) during OWHTO surgery. Pape et al. [19] conducted a cadaveric study on medial joint opening after release of the sMCL. According to their results, the average increase was 3.9 mm after complete release of the sMCL and 3.6 mm after partial release. These increase values (3 mm) were similar to those of the present study. Seo et al. [22] noted that the MJS after fixation with a plate following opening of the osteotomy site (9.2 mm) was significantly decreased compared with the MJS after release of the sMCL (12.2 mm). We speculated as to the reasons why valgus laxity after complete release of the sMCL was significantly decreased by OWHTO. The first reason is tensioning of medial structures (pes anserinus and semimembranosus tendon) other than the sMCL due to medial opening. The second is medial fixation of the locking plate. The third is recovery of valgus laxity after healing of the sMCL after OWHTO. However, Seo et al. did not measure valgus laxity after removal of the locking plate.

OWHTO surgery was performed aiming to MA passing through 65% point lateral from the medial edge of the tibial plateau. However, the mean MA was 69.7% after 2 years. We considered this discrepancy between intra- and post-operative MA in the present study. First, in the OWHTO procedures, intra-operative assessments of alignment have been performed under non-weight bearing conditions with the patient in the supine position. Therefore, discrepancies have arisen between intra-operative prediction of alignment correction and real alignment assessed in standing patients by lower limb radiographs. Second, we have focused on the difference of pre- and postoperative JLCA, which was associated with greater overcorrection of lower limb alignment [31]. Previous study [37] reported that ΔJLCA was significantly correlated with overcorrection. In the present study, the regression analysis also showed moderate correlation between ΔJLCA and overcorrection. From these results, we speculated that amount of change from pre- to postoperative JLCA has affected discrepancies between intra- and postoperative alignment after OWHTO.

Pape et al. [19] reported that complete release of the sMCL during OWHTO has the potential for valgus instability of the knee joint in their biomechanical study. In particular, they mentioned the potential for late valgus instability following OWHTO [19]. Clinical problems regarding valgus instability after OWHTO surgery are very few. However, recently, Kim et al. [38] reported two patients who underwent conversion to total knee arthroplasty (TKA) using a constrained-type implant after OWHTO due to neglected valgus instability. The anatomical changes in the proximal tibia after OWHTO may cause soft tissue imbalance. After OWHTO surgery, the medial tibial plateau is positioned higher than the lateral tibial plateau. If the tibial bone cutting is performed perpendicular to the mechanical axis, it can cause large resection of medial tibial bone that may result in valgus instability and thus increase the risk of soft tissue imbalance. In addition, soft tissue balance should also be considered when performing TKA after OWHTO. Valgus instability can also occur after plate removal during TKA conversion due to the required medial release. Because the distal attachment of the sMCL is usually released in OWHTO, deep MCL release in converting to TKA can affect the biomechanical function of the sMCL, which can cause valgus instability. Therefore, the elevated distal portion of the sMCL should be placed in situ on the graft bone, and pes anserinus, if released, should be repaired during OWHTO to prevent valgus instability. Many surgeons usually release the sMCL subperiosteally distal to the osteotomy site or completely ‘cut’ it at the osteotomy site without repair when performing OWHTO. Therefore, the valgus instability could be the result of the previous OWHTO surgeries.

In the present study, it was noted that complete release of the distal attachment of the sMCL did not cause postoperative valgus instability. There are several reasons why postoperative valgus instability was not observed 2 years after complete release of the MCL during OWHTO. When no bone graft or bone substitute is placed in the osteotomy site in an OWHTO procedure, it is difficult to repair the released sMCL due to keeping the opening space using a lamina spreader, the locking plate is fixed to the medial side of the proximal tibia with screws. In our surgical procedure, the β-TCP spacer was implanted into the opening space. Before locking plate fixation of the tibia, the released sMCL and periosteum above the opening space were repaired. Thus, the medial opening space was covered as possible by the sMCL and periosteum. We speculate that the recovery of valgus laxity might be related to this repair procedure. Although the tension of the sMCL might be increased by the repair, we expected that proximally advancing the sMCL from the original position could prevent increasing medial joint pressure of the knee. Seo et al. [22] also reported that valgus laxity of the knee joint was not observed after OWHTO as in the present study. They concluded that valgus laxity induced by the complete release of the sMCL can be recovered through the tension of the medial structure opening the osteotomy site. These could return the valgus stability level to that before the release of the sMCL. A locking plate is thought to be an important factor to stabilize the knee joint after OWHTO surgery [39]. The present study showed that there were no significant differences in valgus laxity between before surgery and after locking plate removal at 2 years after surgery. However, MRI and histological examinations showed that unstructured and irregularly oriented fibrous tissues were observed in the distal attachment of the sMCL.

There are several limitations to this study. First, the follow-up valgus stress radiography was performed 2 years after surgery. Therefore, at the present time, we cannot speculate on the long-term effect of complete release of the sMCL on valgus stability. Second, the effect of releasing pes anserinus, that is the attachment of the sartorius, gracilis and semitendinosus muscles, was not assessed. Third, it was not clear when the valgus laxity recovered. Stress tests were not performed from after OWHTO surgery to before 1 year after surgery. Fourth, medial joint pressure after tensioning of medial structures due to medial opening was not performed. Therefore, further studies are needed to objectively examine the effects on valgus stability after OWHTO. Fifth, the number of MRI and histological evaluations was limited, which may have caused significant bias.

## Conclusion

This study demonstrated that complete release of the sMCL during OWHTO surgery did not cause postoperative valgus laxity 2 years after the OWHTO procedure.

## Data Availability

The datasets used and analyzed in the current study are available from the corresponding author on reasonable request.

## Abbreviations

JLCA: Joint line convergence angle
MJS: Medial joint space
MRI: Magnetic resonance imaging
OWHTO: Medial open-wedge high tibial osteotomy
sMCL: Superficial layer of the medial collateral ligament
